# HSP90 Inhibition Disrupts 27-Hydroxycholesterol-Induced Inflammatory Signaling in Monocytic Cells

**DOI:** 10.3390/ijms26209963

**Published:** 2025-10-13

**Authors:** Jaesung Kim, Munju Kwon, Dongha Park, Nakyung Kang, Yonghae Son, Ninib Baryawno, Byoung Soo Kim, Sik Yoon, Sae-Ock Oh, Dongjun Lee, Koanhoi Kim

**Affiliations:** 1 Department of Pharmacology, School of Medicine, Pusan National University, Yangsan 50612, Republic of Korea; rlawotjd1029@naver.com (J.K.); propertopic@pusan.ac.kr (D.P.); ka776@naver.com (N.K.); squall0211@hanmail.net (Y.S.); 2 Department of Convergence Medicine, School of Medicine, Pusan National University, Yangsan 50612, Republic of Korea; gmj0226@naver.com; 3 Childhood Cancer Research Unit, Department of Women’s and Children’s Health, Karolinska Institute, 17177 Stockholm, Sweden; n.baryawno@ki.se; 4 School of Biomedical Convergence Engineering, Pusan National University, Yangsan 50612, Republic of Korea; bskim7@pusan.ac.kr; 5 Department of Anatomy, School of Medicine, Pusan National University, Yangsan 50612, Republic of Korea; sikyoon@pusan.ac.kr (S.Y.); hedgehog@pusan.ac.kr (S.-O.O.); 6 Transplantation Research Center, Research Institute for Convergence of Biomedical Science and Technology, Pusan National University Yangsan Hospital, Yangsan 50612, Republic of Korea

**Keywords:** Akt/mTORC1, heat shock protein 90, 27-hydroxycholesterol, inflammation, monocytic cell activation

## Abstract

27-Hydroxycholesterol (27OHChol), a cholesterol metabolite, induces inflammatory responses in monocytic cells and promotes their differentiation into mature dendritic cells. Here, we examined whether inhibition of heat shock protein 90 (HSP90) modulates these responses. Treatment with ganetespib, a selective HSP90 inhibitor, significantly reduced chemokine CCL2 expression, lowering monocytic cell migration. It also suppressed matrix metalloproteinase-9 (MMP-9) expression and attenuated the lipopolysaccharide (LPS) response otherwise amplified by 27OHChol. Furthermore, ganetespib decreased mature dendritic cell markers (CD80, CD83, CD88) and restored endocytic activity, indicating a less activated state. These changes suggest that HSP90 regulates 27OHChol-induced pro-inflammatory activation via its client proteins. To explore this mechanism, we examined the phosphorylation status of signaling proteins. 27OHChol enhanced phosphorylation of Akt and its downstream targets, S6 and 4E-BP1 within the Akt/mTORC1 pathway. Ganetespib reduced total and phosphorylated Akt and 4E-BP1, and selectively inhibited S6 phosphorylation without altering total protein level. Collectively, these findings demonstrate that HSP90 inhibition by ganetespib mitigates 27OHChol-driven monocytic cell activation through suppression of the HSP90-Akt/mTORC1 axis. Targeting this pathway may provide a promising therapeutic strategy for metabolic inflammation associated with oxysterols.

## 1. Introduction

Low-grade chronic inflammation is a hallmark of numerous pathological conditions, including cardiovascular disease, and is often exacerbated by lipid accumulation and oxidative stress [1,2]. Among the various oxidized cholesterol derivatives, 27-hydroxycholesterol (27OHChol) has emerged as a key mediator of chronic inflammation. Elevated levels of 27OHChol have been detected in atherosclerotic lesions [3], where it contributes to vascular inflammation and promotes monocyte/macrophage infiltration, an essential step in the development of atherosclerotic plaques. Beyond its role in cardiovascular disease, 27OHChol is also implicated in the dysregulation of lipid metabolism and the development of insulin resistance, underscoring its broader involvement in the pathogenesis of metabolic syndromes [4,5]. From the cellular perspective, 27OHChol stimulates monocytic cells to express pro-inflammatory chemokines including CCL2, which plays a critical role in recruiting monocytes [6]. 27OHChol also enhances cellular responses to Toll-like receptor agonists [7,8] and promotes the differentiation of monocytic cells into phenotypically mature dendritic cells (mDCs) [9], further amplifying immune responses. Despite these findings, the molecular mechanisms underlying 27OHChol-induced inflammation and the regulation of these inflammatory responses remain incompletely understood.

A potential mechanism through which 27OHChol exerts its biological effects is via modulation of heat shock proteins (HSPs), as indicated by prior studies [10,11]. HSPs are molecular chaperones that facilitate proper protein folding, stabilize misfolded or aggregated proteins, and assist in transmembrane transport [12,13]. They also play essential roles in cellular signaling by facilitating the formation of protein complexes involving key signaling molecules, such as mammalian target of rapamycin (mTOR) and serine/threonine kinase B (Akt) [14]. Among the HSPs, HSP90 is particularly important because of its ability to stabilize and regulate a wide range of client proteins involved in cell survival and inflammatory responses, including components of the PI3K/Akt/mTOR pathway [15]. Pharmacological inhibition of HSP90 has been demonstrated to suppress the expression of pro-inflammatory molecules and attenuate monocytic cell and T-cell migration in both in vitro and in vivo models [16,17]. Owing to its capacity to interfere with multiple signaling proteins implicated in stress and inflammatory responses, HSP90 inhibition has emerged as a promising therapeutic approach for modulation of autoimmune and inflammatory diseases [18].

Several studies have identified distinct chemical compounds that inhibit or impair cellular responses induced by 27OHChol [19,20,21]. Nevertheless, there remains a significant gap in the development of drugs specifically targeting inflammation mediated by oxidized cholesterol derivatives, particularly with respect to HSPs. Given the established role of HSP90 in inflammatory pathways, the present study aimed to evaluate the efficacy of ganetespib (also known as STA9090), a triazolone compound that competitively binds to the N-terminal ATP-binding domain of HSP90, thereby inhibiting its chaperone activity [22,23], in mitigating 27OHChol-induced inflammatory responses. Using the THP-1 cell line, a widely accepted model for studying monocytic cells [24], we investigated the effects of ganetespib on multiple inflammatory parameters. Specifically, we assessed its impact on CCL2 production, monocyte migration, matrix metalloproteinase-9 (MMP-9) expression, and modulation of the mTORC1 signaling pathway. Furthermore, we evaluated the influence of ganetespib on the expression of markers associated with mature dendritic cells (mDCs). Our findings offer novel insights into the role of HSP90 in oxysterol-induced inflammation and suggest that targeting HSP90 may constitute a viable therapeutic strategy for diseases characterized by excessive monocyte recruitment and activation within oxysterol-enriched microenvironments.

## 2. Results

### 2.1. Ganetespib Decreases 27OHChol-Induced CCL2 Production and Monocytic Cell Migration

**Figure 1:**
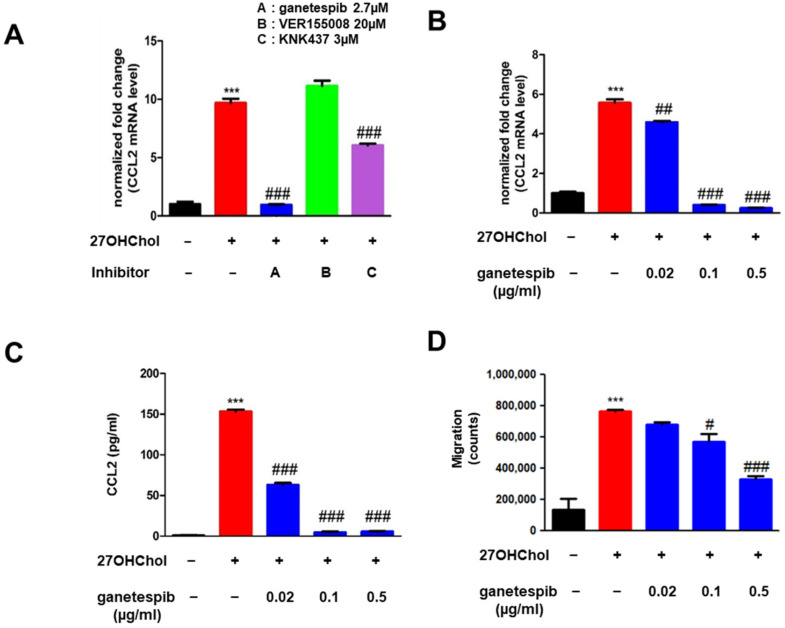
Effects of ganetespib on 27OHChol-induced CCL2 expression and monocytic cell migration. THP-1 cells were treated for 48 h with 27OHChol (2 μg/mL) in the presence of the indicated HSP90 inhibitors (**A**) or the indicated concentrations of ganetespib (**B**). CCL2 mRNA expression levels were quantified using real-time PCR. Data are presented as the mean ± SD, with three independent replicates per group. (**C**) Following stimulation of THP-1 cells with 27OHChol in combination with ganetespib, the culture supernatants were harvested, and the amount of secreted CCL2 protein was measured by ELISA. Data are presented as the mean ± SD, with three independent replicates per group. (**D**) Monocytic cells’ migration was evaluated by chemotaxis experiment using the supernatants isolated after stimulation with 27OHChol in the presence of ganetespib. Data are presented as the mean ± SD, with three independent replicates per group. *** *p* < 0.001 compared to control; # *p* < 0.05 compared to 27OHChol; ## *p* < 0.01 compared to 27OHChol; ### *p* < 0.001 compared to 27OHChol.

To identify compounds capable of attenuating 27OHChol-induced inflammatory responses, we screened a panel of HSP inhibitors. THP-1 cells were serum-starved for 24 h, followed by incubation for 48 h with 27OHChol in the presence or absence of the indicated inhibitors. Ganetespib (HSP90 inhibitor), VER155008 (HSP70 inhibitor), and KNK437 (HSP70 inhibitor) were applied to the cells at concentrations of 2.7 µM, 20 µM, and 3 µM, respectively, each corresponding to 1 μg/mL (Figure 1A and Supplementary Figure S1). The CCL2 transcript levels and secretion of CCL2 protein were profoundly decreased in cells treated with ganetespib. The decrease was not attributable to cytotoxicity as ganetespib did not alter cell viability (Supplementary Figure S2). We carried out an experiment to determine whether ganetespib alone affects the expression of CCL2 and MMP9 in monocytes, and found that it had no significant effect when administered as a single agent (Supplementary Figure S3). A reduction was also observed with KNK437, to a smaller extent, compared to that of ganetespib. VER155008, however, did not change CCL2 expression. Therefore, we decided to focus primarily on ganetespib in the subsequent experiment.

27OHChol treatment significantly elevated CCL2 mRNA levels, while ganetespib at concentrations of 0.02, 0.1, and 0.5 µg/mL caused a dose-dependent decrease (Figure 1B). Similarly, the secretion of CCL2 by THP-1 cells was markedly increased following 27OHChol treatment compared to unstimulated controls. However, the addition of ganetespib to 27OHChol-treated THP-1 cells resulted in a concentration-dependent reduction in CCL2 secretion (Figure 1C). To investigate the effect of ganetespib on monocytic cell migration, a chemotaxis assay was performed. 27OHChol treatment enhanced THP-1 cell migration, but this effect was attenuated in a dose-dependent manner by ganetespib (Figure 1D). Cell migration was reduced in the group that was exposed to supernatant isolated in the presence of 0.1 and 0.5 µg/mL of ganetespib. These findings demonstrate that ganetespib suppresses both CCL2 production and monocyte migration induced by 27OHChol.

### 2.2. Ganetespib Suppresses the Superinduction of CCL2

**Figure 2:**
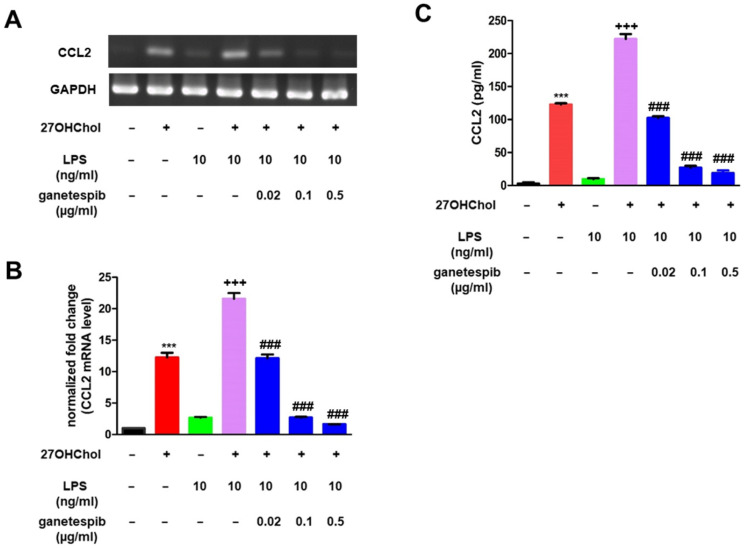
Impaired superinduction of CCL2 in the presence of ganetespib. THP-1 cells stimulated with 27OHChol (2 μg/mL) for 24 h were treated with LPS in the presence of ganetespib. (**A**) CCL2 transcripts were qualitatively presented as band images using RT-PCR. (**B**) CCL2 mRNA expression levels were quantified using real-time PCR. Data are presented as the mean ± SD, with three independent replicates per group. (**C**) The culture supernatants were harvested, and the amount of secreted CCL2 protein was measured by ELISA. Data are presented as the mean ± SD, with three independent replicates per group. *** *p* < 0.0001 compared to control; ### *p* < 0.0001 compared to 27OHChol; +++ *p* < 0.0001 compared to 27OHChol with LPS.

The addition of LPS to 27OHChol-stimulated cells further enhanced CCL2 production, a process referred to as superinduction [7]. To investigate the effects of ganetespib on this enhanced response, real-time PCR and ELISA were performed (Figure 2). CCL2 transcript levels were elevated following 27OHChol treatment, with a further increase observed upon LPS stimulation. However, ganetespib administration resulted in a dose-dependent suppression of this induction (Figure 2A,B). The secretion of CCL2, which was elevated by 27OHChol and further augmented by LPS, was effectively reduced by ganetespib, consistent with the observed decrease in gene transcription (Figure 2C). These findings demonstrate that ganetespib effectively inhibits CCL2 expression in monocytes activated by LPS in combination with 27OHChol.

### 2.3. Ganetespib Suppresses the Expression of MMP-9 Induced by 27OHChol

**Figure 3:**
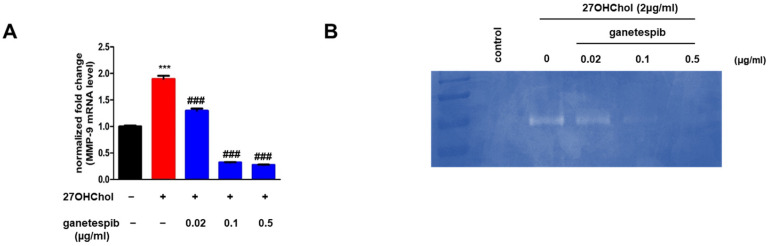
Effects of ganetespib on 27OHChol-induced MMP-9 expression. THP-1 cells were stimulated with 27OHChol (2 μg/mL) in the absence or presence of ganetespib. (**A**) MMP-mRNA expression levels were quantified using real-time PCR. Data are presented as the mean ± SD, with three independent replicates per group. (**B**) The culture supernatants were harvested, and the activity of MMP-9 was evaluated by gelatin zymography *** *p* < 0.0001 compared to control; ### *p* < 0.0001 compared to 27OHChol.

Treatment of THP-1 cells with 27OHChol induced the production of MMP-9, an enzyme produced by immune cells such as monocytes, which is essential for the migration of immune cells to sites of inflammation. Accordingly, we investigated the effect of ganetespib on MMP-9 synthesis and secretion (Figure 3). 27OHChol treatment significantly elevated MMP-9 mRNA levels, while ganetespib administration at concentrations of 0.02, 0.1, and 0.5 µg/mL caused a dose-dependent decrease in MMP-9 expression (Figure 3A). To further assess the activity of MMP-9 secreted by THP-1 cells, gelatin zymography was performed. Treatment with 27OHChol increased MMP-9 activity, whereas ganetespib treatment reduced its activity in a dose-dependent manner (Figure 3B). These findings suggest that ganetespib inhibits both the synthesis and activity of MMP-9 induced by 27OHChol.

### 2.4. Ganetespib Influences 27OHChol-Induced Maturation and Functional Changes in Monocytes

**Figure 4:**
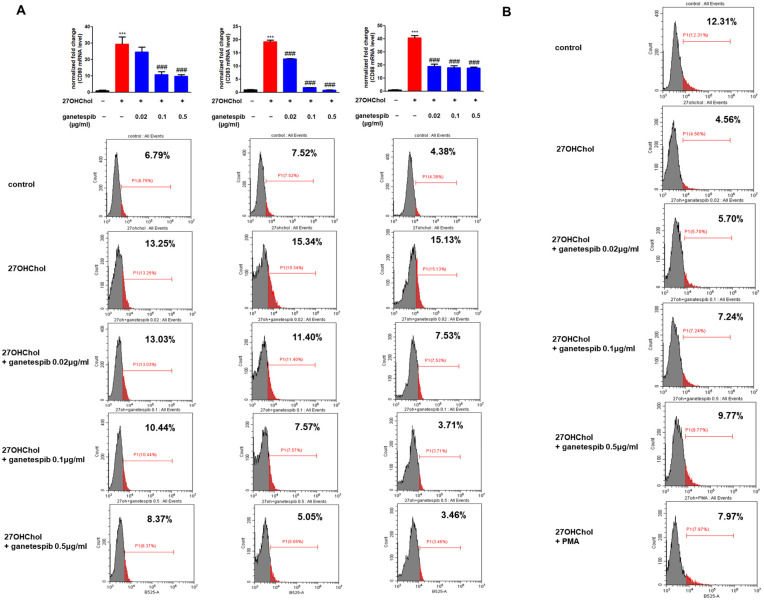
Expression of mDC markers and endocytic function. THP-1 cells were stimulated with 27OHChol (2 μg/mL) in the absence or presence of ganetespib for 48 h. (**A**) mRNA expression levels of CD80, CD83, and CD88 were quantified using real-time PCR. Data are presented as the mean ± SD, with three independent replicates per group. *** *p* < 0.0001 compared to the control; ### *p* < 0.0001 compared to 27OHChol. Expression of CD80, CD83, and CD88 was further examined by flow cytometry after staining the cells with FITC-conjugated antibodies. The data presented are representative of three independent experiments. (**B**) Following treatment of THP-1 cells with 27OHChol (2 µg/mL) alone or in combination with the indicated concentrations of ganetespib for 48 h, cells were incubated with FITC-conjugated dextran for 30 min and subsequently analyzed by flow cytometry. As a positive control, cells were co-treated with 27OHChol and PMA (250 nM). The data presented are representative of three independent experiments.

We examined the effects of ganetespib on 27OHChol-induced maturation and functional changes in monocytes. The maturation and functional changes were assessed using the representative mDC markers CD80, CD83, and CD88. Upon treatment with 27OHChol, quantitative PCR analysis revealed a significant increase in the transcription levels of the mDC markers CD80, CD83, and CD88 compared to the control group. However, this upregulation was inhibited in a concentration-dependent manner upon treatment with ganetespib (Figure 4A). Furthermore, flow cytometry analysis demonstrated that 27OHChol treatment significantly enhanced the surface expression of CD80, CD83, and CD88, whereas ganetespib treatment led to a dose-dependent reduction in their expression (Figure 4A). These findings suggest that ganetespib inhibits both the transcription and surface expression of mDC markers induced by 27OHChol, thereby impacting the maturation and functional changes of monocytes driven by 27OHChol.

We conducted an endocytosis assay to evaluate whether ganetespib could reverse the functional alterations induced by 27OHChol in monocytic cells (Figure 4B). Treatment with 27OHChol led to a marked reduction in endocytic function compared to the control group. However, this impaired function was dose-dependently restored upon ganetespib treatment. Furthermore, co-treatment with 27OHChol and phorbol myristate acetate (PMA), which served as positive controls, restored endocytic function. These findings suggest that ganetespib effectively reverses the endocytic impairment caused by 27OHChol in monocytic cells.

### 2.5. Ganetespib Affects Phosphorylation of Downstream Protein of mTORC1 Signaling Pathway Increased in the Presence of 27OHChol

**Figure 5:**
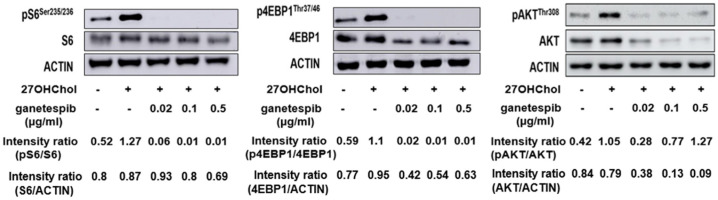
Immunoblot analysis of proteins involved in upstream and downstream pathways of mTORC1 signaling. THP-1 cells (2.5 × 10^5^ cells/mL) were serum-starved for 24 h and subsequently treated with 27OHChol together with 0.02, 0.1, or 0.5 μg/mL ganetespib for 48 h, after which phosphorylated and unphosphorylated forms of S6, 4E-BP1, and AKT were detected by immunoblotting.

Stimulation with 27OHChol led to increased phosphorylation of Akt, as well as its downstream targets S6 and 4E-BP1, indicating activation of the Akt/mTORC1 signaling pathway (Figure 5). To assess whether HSP90 contributes to this pathway, we examined the effects of prolonged HSP90 inhibition (Figure 5). Treatment with ganetespib for 48 h at concentrations of 0.02, 0.1, or 0.5 μg/mL did not affect total S6 protein levels; however, phosphorylation of S6 was markedly reduced. In contrast, both total and phosphorylated levels of Akt and 4E-BP1 were decreased in a concentration-dependent manner. This suggests that prolonged HSP90 inhibition downregulates these proteins, likely through destabilization of client proteins such as Akt. Taken together, these findings indicate that 27OHChol activates the Akt/mTORC1 pathway, and that HSP90 activity is required to sustain this activation, possibly by maintaining the stability and phosphorylation of key signaling components.

## 3. Discussion

Our results demonstrate the efficacy of HSP90 inhibition in mitigating inflammatory responses and functional impairments induced by 27OHChol in monocytic cells. Treatment with ganetespib significantly decreased the production of CCL2 and restored endocytic function in these cells. Notably, the superinduction of CCL2 observed following combined treatment with LPS and 27OHChol was also significantly suppressed by ganetespib. This indicates that ganetespib not only reduces baseline CCL2 production but also attenuates the exaggerated inflammatory response elicited by additional stimuli. The reduction in both CCL2 transcript levels and protein secretion after ganetespib treatment highlights its ability to modulate the inflammatory cascade at multiple levels. In particular, ganetespib suppressed 27OHChol-induced CCL2 and TNF-α at both the transcript and protein levels in a dose-dependent manner (Supplementary Figure S4), underscoring its importance in limiting monocyte recruitment to inflamed tissues and the progression of inflammatory diseases. [25,26]. Furthermore, ganetespib effectively blocked 27OHChol-induced monocytic cell migration, suggesting its potential to prevent monocyte infiltration into inflamed tissues. In contrast, the reduction in CCL2 levels observed with ganetespib was not replicated by the HSP70 inhibitor VER155008 [27,28]. Treatment with KNK437, which inhibits the induction of multiple heat shock proteins including HSP105, HSP70, and HSP40 [29,30], also reduced CCL2 levels; however, this effect was markedly less pronounced compared to ganetespib. These findings underscore the specific and potent role of HSP90 inhibition in regulating 27OHChol-induced inflammatory responses in monocytic cells.

The present study also demonstrates that ganetespib downregulates matrix metalloproteinase-9 (MMP-9), an enzyme critically involved in extracellular matrix remodeling and macrophage infiltration [31,32]. The observed dose-dependent decrease in both MMP-9 transcript levels and enzymatic activity suggests that ganetespib can modulate tissue remodeling processes associated with chronic inflammation and atherosclerosis. This modulatory effect may prove particularly beneficial in pathological conditions where MMP-9 plays a key role in disease progression. Importantly, our endocytosis assays revealed that ganetespib restores the endocytic function of monocytic cells impaired by 27OHChol. The recovery of endocytic activity is essential for maintaining normal cellular function and an effective immune response [33]. The dose-dependent restoration of endocytic function by ganetespib further underscores its potential to reverse functional impairments in immune cells induced by pro-inflammatory stimuli.

HSP90 plays a crucial role in stabilizing and activating several client proteins, including those involved in key signaling pathways [34]. One of the critical pathways regulated by HSP90 is the mammalian target of rapamycin complex 1 (mTORC1) signaling pathway. The relationship between HSP90 and mTORC1 is multifaceted. HSP90 stabilizes key components of the mTORC1 complex, thereby maintaining its functional integrity and enabling appropriate responses to cellular cues such as nutrient availability and stress signals [35]. Under stress conditions, HSP90 further contributes to the reactivation of mTORC1 by regulating stress-associated signaling pathways, thus facilitating cellular adaptation to environmental challenges [36]. Inhibition of HSP90 disrupts the stability and activity of the mTORC1 complex, leading to downstream alterations in cell metabolism and growth. In our study, ganetespib treatment resulted in an increased pAkt/AKT ratio; however, this was attributable to a reduction in total AKT levels rather than an increase in phosphorylation. These findings suggest that ganetespib downregulates AKT expression by impairing HSP90-dependent protein stabilization.

Further analysis revealed that ganetespib suppresses the mTORC1 signaling pathway, as evidenced by decreased phosphorylation of downstream effectors S6 and 4E-BP1. As shown in Supplementary Figure S5, ganetespib downregulates AKT, S6, and 4E-BP1 in the presence of elevated 27OHChol, consistent with inhibition of mTORC1 signaling. Given that AKT functions upstream, and S6 and 4E-BP1 downstream, within the mTORC1 axis, these findings collectively support the conclusion that ganetespib suppresses mTORC1 activity. This suppression has functional consequences: reduced activation of the mTORC1 pathway correlates with decreased transcription of inflammatory mediators, such as CCL2 and MMP-9, ultimately leading to attenuated monocyte migration and inflammatory responses. These mechanistic insights help explain ganetespib’s anti-inflammatory effects. A comprehensive summary of these findings is provided in Supplementary Table S3, highlighting the comparative impact of ganetespib on key inflammatory parameters.

Collectively, these findings indicate that ganetespib is a potent inhibitor of 27OHChol-induced inflammatory responses in monocytic cells. It significantly reduces CCL2 production, inhibits monocytic cell migration, downregulates MMP-9 expression, and restores endocytic function. These effects underscore its therapeutic potential for treating inflammatory conditions characterized by excessive monocyte-driven immune responses. Future studies should focus on elucidating the detailed molecular mechanisms through which ganetespib modulates these inflammatory pathways, particularly in the context of in vivo models. In addition, exploring potential synergistic effects between ganetespib and other anti-inflammatory agents may provide valuable insights for the development of more effective combination therapies. In conclusion, HSP90 inhibition represents a promising strategy for modulating inflammation and restoring immune cell function in pathological conditions driven by cholesterol derivatives such as 27OHChol.

## 4. Materials and Methods

### 4.1. Reagents

Ganetespib, VER-155008 and KNK437 were purchased from MedChemExpress (Monmouth Junction, NJ, USA). 27OHChol was obtained from Sigma-Aldrich (St. Louis, MO, USA). LPS extracted from *Escherichia coli* K12 was acquired from InvivoGen (San Diego, CA, USA). Antibodies against S6 ribosomal protein, Akt (pan), and their phosphorylated forms were sourced from Cell Signaling Technology, Inc. (Danvers, MA, USA). Antibodies specific to β-actin, 4E-BP1, and phosphorylated 4E-BP1 were obtained from Santa Cruz Biotechnology, Inc. (Santa Cruz, CA, USA).

### 4.2. Cell Culture and Treatment

THP-1 cells were obtained from the American Type Culture Collection (ATCC, Manassas, VA, USA) and cultured in RPMI 1640 medium supplemented with 10% fetal bovine serum (FBS), penicillin (100 IU/mL), and streptomycin (10 µg/mL) at 37 °C in a 5% CO_2_ incubator. Cells were maintained in T25 flasks (SPL, Gyeonggi-do, Republic of Korea) and sub-cultured every 48 h. Prior to treatment, the cells were serum-starved overnight in RPMI 1640 medium containing 0.1% bovine serum albumin (BSA; GenDEPOT, Katy, TX, USA). They were then treated with 27OHChol (2 µg/mL) and/or ganetespib at various concentrations. 27OHChol was dissolved in ethanol, and ganetespib was dissolved in dimethyl sulfoxide.

### 4.3. Reverse Transcription PCR (RT-PCR)

After cell lysis with TRIzol reagent (Molecular Research Center, Cincinnati, OH, USA), chloroform was added to isolate the aqueous phase containing RNA, which was then separated by centrifugation. RNA was precipitated with isopropyl alcohol, and the resulting pellet was washed with 70% ethanol. RNA concentration was quantified using a NanoDrop spectrophotometer. After normalization of RNA concentrations, cDNA was synthesized using reverse transcriptase (Promega, Madison, WI, USA), followed by PCR amplification with DNA polymerase for 25 cycles. A 6× loading dye was added to each sample, and the samples were loaded onto a 2% agarose gel for electrophoresis. The gels were stained with ethidium bromide for 30 min, and bands were visualized using the WSE-5300 PrintGraph CMOS I (Tokyo, Japan). Quantitative PCR was performed with SYBR Green PCR Master Mix, with all samples run in triplicate. Relative gene expression was calculated using the 2^−ΔΔCt^ method, with transcript levels normalized to GAPDH expression. The primers used in this study are detailed in the Supplementary Tables.

### 4.4. Western Blot Analysis

Cells were lysed using Pro-prep protein extraction buffer. Protein concentration was then quantified using the BCA assay (Thermo Fisher Scientific, Waltham, MA, USA) with an albumin standard. After quantification, samples were mixed with 5× SDS loading buffer and heated at 95 °C. A uniform amount (20 µg) of protein was subsequently loaded onto a 12% SDS-PAGE gel for electrophoretic separation. The separated proteins were transferred onto a nitrocellulose (NC) membrane and subsequently blocked with 5% skim milk for 1 h to prevent nonspecific binding. The membrane was then incubated with primary antibodies diluted in 5% skim milk overnight at 4 °C. After three 15-min washes with TBS-T, the membranes were incubated with HRP-conjugated secondary antibodies diluted in TBS-T at room temperature for 1 h, followed by an additional three 15-min washes with washing buffer. The HRP substrate was applied, and protein bands were visualized using chemiluminescence reagents. Detection was performed with the Amersham Imager 680 system.

### 4.5. Enzyme-Linked Immunosorbent Assay (ELISA)

After cell harvest, samples were centrifuged to separate the supernatant, which was subsequently filtered through a syringe filter. CCL2 levels in the supernatant were measured using an ELISA kit (Human MCP-1 ELISA kit; BD Biosciences, San Diego, CA, USA). Standards and samples were added to the designated wells, and the plate was incubated at room temperature for 2 h. Following this, the wells were washed five times with wash buffer. The working detector was then added to each well, and the plate was incubated for an additional hour at room temperature. After a second wash with wash buffer, substrate solution was introduced into each well, followed by a 30 min incubation in the dark at room temperature. Finally, a stop solution (2N sulfuric acid) was added, and absorbance was measured at 450 nm.

### 4.6. Cell Migration Assay

Transwell permeable supports (Costar, Cambridge, MA, USA) were employed to assess the migratory response of THP-1 cells. Transwell inserts were placed into wells containing supernatants enriched with CCL2, which had been harvested following the treatment of cells with 27OHChol and ganetespib. Serum-starved THP-1 cells (2.5 × 10^5^) suspended in RPMI 1640 medium supplemented with 0.1% BSA were added to the upper chamber of polycarbonate Transwell inserts with a 5 μm pore size. After incubation at 37 °C in a 5% CO_2_ atmosphere for 2 h, the upper chamber was removed. Cell viability was subsequently assessed using a Vi-Cell cell counter (Beckman Coulter, Inc., Brea, CA, USA) following the addition of trypan blue solution. Non-viable cells exhibited blue staining, while viable cells remained unstained.

### 4.7. MMP-9 Gelatin Zymography

After cell harvest, centrifugation was conducted to isolate the supernatant, which was subsequently filtered through a syringe filter and concentrated 20-fold using a Vivaspin 2 Centricon (Sartorius, Göttingen, Germany). Electrophoresis was then carried out on ice at 50 V for 4 h using an 8% gel containing 2% gelatin. Following electrophoresis, the gel was washed twice in washing buffer, with each wash lasting 30 min. The gel was then incubated in incubation buffer at 37 °C for 20 h. After incubation, it was stained with Coomassie Brilliant Blue R-250 (Bio-Rad Laboratories, Hercules, CA, USA) and destained. The results were visualized using the WSE-5300 PrintGraph CMOS I (Tokyo, Japan).

### 4.8. Flow Cytometry

Following cell harvesting and washing with PBS, the cells were incubated with FITC-conjugated dextran or antibodies specific to CD80, CD83, and CD88 at a 1:100 dilution for 4 h. After incubation, the cells were washed, resuspended in PBS, and analyzed via flow cytometry (Beckman Coulter, Inc., Brea, CA, USA).

### 4.9. Statistical Analysis

Data are presented as mean ± SD. Statistical analyses were conducted using PRISM software (version 5.0; GraphPad Software Inc., San Diego, CA, USA). One-way ANOVA followed by Dunnett’s multiple comparison test was applied. A *p*-value < 0.05 was considered statistically significant.

## Data Availability

The raw data supporting the conclusions of this article will be made available by the corresponding authors on request.

## Supplementary Materials

The following supporting information can be downloaded at: https://www.mdpi.com/article/10.3390/ijms26209963/s1.
