# Supplementary material for: HSP90 Inhibition Disrupts 27-Hydroxycholesterol-Induced Inflammatory Signaling in Monocytic Cells
Source: Int J Mol Sci. 2025 Oct 13;26(20):9963. doi: 10.3390/ijms26209963 (PMC12563181; doi:10.3390/ijms26209963)
Supplement: Supplementary file 1 [file ijms-26-09963-s001.zip › ijms-3860446-supplementary.pdf]

Supplementary Figure S1

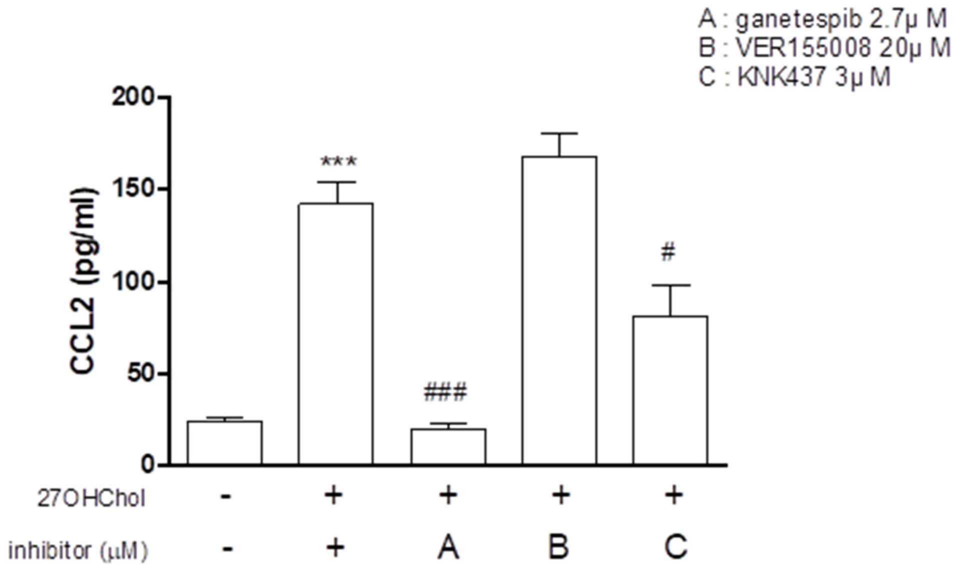

Figure S1. Effects of inhibitors on CCL2 secretion in monocytic cell.

THP-1 cells were treated with 27OHChol and the indicated concentrations of inhibitors for 48 h. Secretion level of CCL2 were quantified using ELISA kit. Data are presented as the mean ± SD, with three independent replicates per group. ***P < 0.001 compared to control; # P < 0.05, ### P < 0.001 compared to 27OHChol.

Supplementary Figure S2

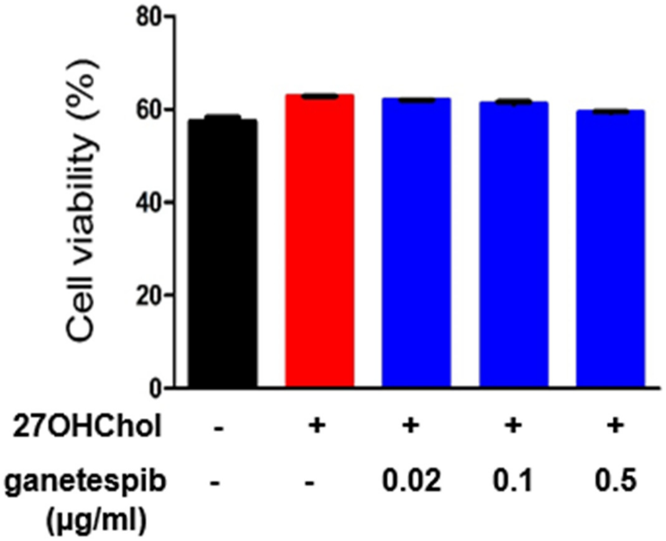

Figure S2. Cell viability following treatment with ganetespib.

The serum-starved THP-1 cells were treated with 27OHChol in the presence or absence of ganetespib at concentrations of 0.02, 0.1, and 0.5 µg/mL for 48 h. Cell viability was measured using a Vi-Cell cell counter (Beckman Coulter, Inc., Brea, CA).

Supplementary Figure S3

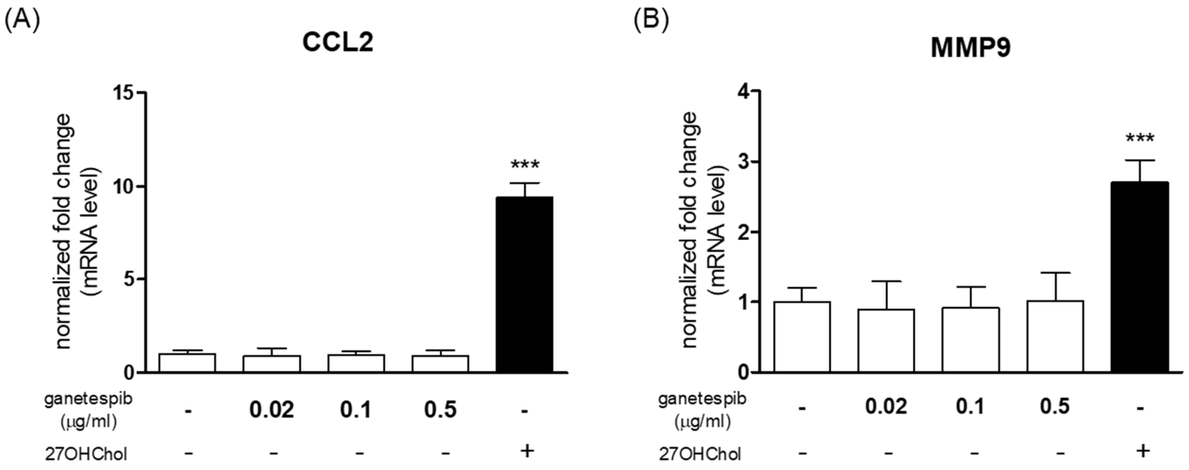

Figure S3. Effects of ganetespib on CCL2 and MMP9 expression in monocytic cell.

THP-1 cells were treated with the indicated concentrations of ganetespib for 48 h. CCL2 (A) and MMP9 (B) mRNA expression levels were quantified using real-time PCR. 27OHChol served as a positive control in the experiments. Data are presented as the mean ± SD, with three independent replicates per group. ***P < 0.001 compared to control.

Supplementary Figure S4

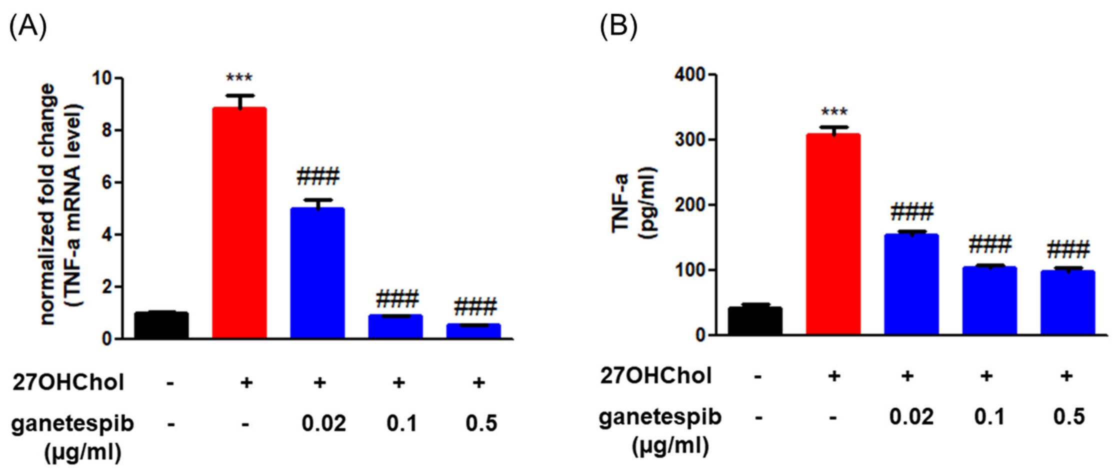

Figure S4. Effects of ganetespib on TNF-α expression in monocytic cell.

THP-1 cells were treated with the indicated concentrations of ganetespib for 48 h. TNF-α mRNA expression levels were quantified using real-time PCR (A) and the amount of secreted TNF-α protein was measured by ELISA (B). 27OHChol served as a positive control in the experiments. Data are presented as the mean ± SD, with three independent replicates per group. ***P < 0.001 compared to control.

Supplementary Figure S5

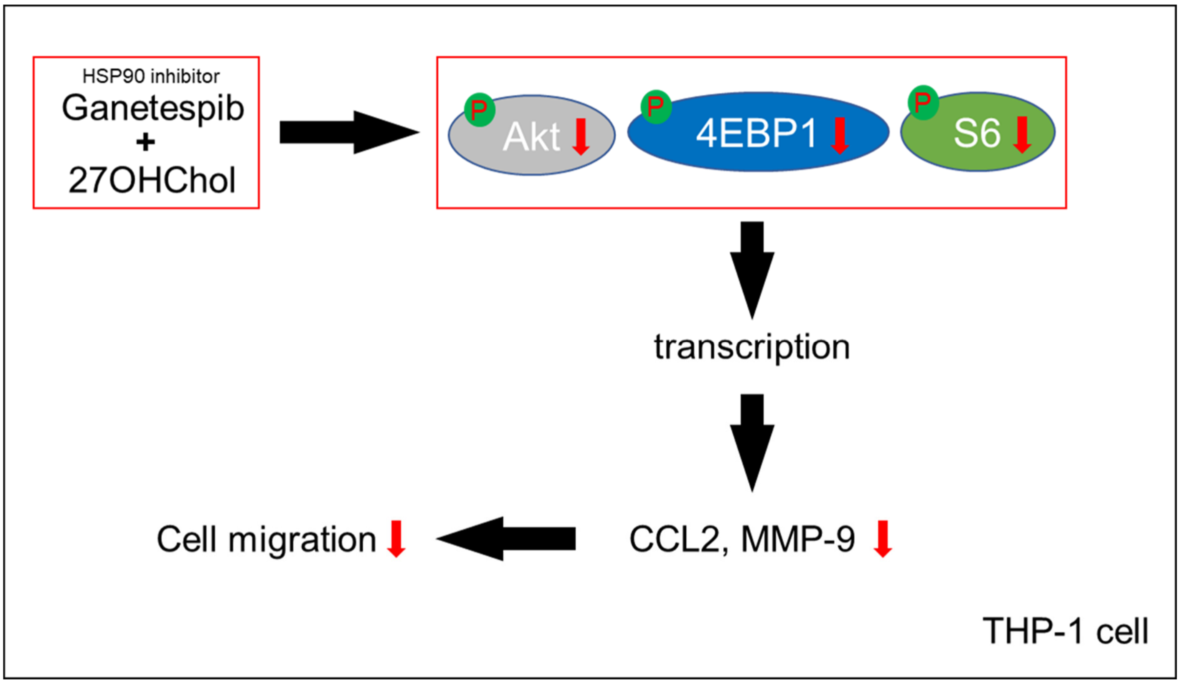

Figure S5. Downstream effects of ganetespib-mediated inhibition of Akt, 4EBP1, and S6 signaling, resulting in reduced CCL2 and MMP-9 expression and impaired THP-1 cell migration.

Table S1. Non-quantitative PCR primer sequences evaluated in this study were as follows:

Primer sequences for non-quantitative PCR	
Primers	Sequences
Human GAPDH	Forward 5’–GAGTCAACGGATTTGGTCCT–3’
	Reverse 5’–TGTGGTCATGAGTCCTTCCA–3’’
Human CCL2	Forward 5’–TCTGTGCCTGCTGCTCATAG–3’
	Reverse 5’–CAGATCTCCTTGGCCACAAT–3’

Table S2. Quantitative real-time PCR primer sequences evaluated in this study were as follows:

Primer sequences for real-time PCR	
Primers	Sequences
Human GAPDH	Forward 5'–GAAGGTGAAGGTCGGAGT–3'<br>Reverse 5'–GAAGATGGTGATGGGATTTC–3'
Human CCL2	Forward 5'–CAGCCAGATGCAATCAATGCC–3'<br>Reverse 5'–TGGAATCCTGAACCCACTTCT–3'
Human CD80	Forward 5'–GCAGGGAACATCACCATCCA-3'<br>Reverse 5'–TCACGTGGATAACACCTGAACA-3'
Human CD83	Forward 5'–TCCTGAGCTGCGCCTACAG-3'<br>Reverse 5'–GCAGGGCAAGTCCACATCTT-3'
Human CD88	Forward 5'–GTGGTCCGGGAGGAGTACTTT-3'<br>Reverse 5'–GCCGTTTGTCGTGGCTGTA-3'
Human MMP-9	Forward 5'–GCACGACGTCTTCCAGTACC-3'<br>Reverse 5'–CAGGATGTCATAGGTCACGTAGC-3'

Table S3. Summary of Ganetespib's Effects on Key Inflammatory Markers and Cell Migration:

Measured Variable	Effects of 27OHChol	Effects of Ganetespib
CCL2 (mRNA expression levels)	Significant upregulation	Dose-dependent decrease
CCL2 protein secretion	Significant increase	Dose-dependent decrease
Monocyte migration	Significantly enhanced	Dose-dependent reduction
MMP-9 (mRNA expression levels)	Significant upregulation	Dose-dependent decrease
